# Resveratrol supplementation improves uterine immune resolution in mares susceptible to persistent breeding-induced endometritis

**DOI:** 10.3389/fvets.2026.1760273

**Published:** 2026-02-16

**Authors:** Genevieve Denison, Javier Funes, Patricio Razquin, Jocelyn Howard, Isabela Hamner, Lyta Foulk, Chelsie Burden, Jenn N. Hatzel, Jenny L. Sones, Pat M. McCue, Carleigh E. Fedorka

**Affiliations:** 1 Department of Animal Sciences, Colorado State University, Fort Collins, CO, United States; 2 Department of Clinical Sciences, Colorado State University, Fort Collins, CO, United States; 3 Department of Biomedical Sciences, Colorado State University, Fort Collins, CO, United States

**Keywords:** cytokine, neutrophil, PBIE (persistent breeding-induced endometritis), resveratrol, uterine inflammation

## Abstract

**Introduction:**

Persistent breeding-induced endometritis (PBIE) remains a major problem in equine reproduction. This disorder is characterized by prolonged post-breeding inflammation and is associated with advanced age, reduced endometrial quality, and impaired uterine immune function. Resveratrol, a polyphenolic compound with documented anti-inflammatory activity in humans, has not been investigated for its ability to modulate uterine inflammation in mares. We hypothesized that resveratrol supplementation would attenuate the exaggerated inflammatory response characteristic of PBIE-susceptible mares.

**Methods:**

Six susceptible mares completed a control cycle followed by 21.8 ± 0.8 days of oral resveratrol treatment (2,800 g SID). During both cycles, mares were inseminated with 500 × 106 freezekilled spermatozoa and evaluated at 6 and 24 h post-breeding. Uterine fluid was quantified via transrectal ultrasonography, and neutrophil counts were assessed using cytobrush and low-volume lavage (LVL). Luminal cytokines were measured with an equine multiplex immunoassay. Data were analyzed using a general linear model in SAS 9.4® (*p* ≤ 0.05).

**Results:**

Resveratrol supplementation did not alter uterine fluid accumulation (*p* = 0.35) but reduced neutrophilia in LVL samples at 6 h (*p* = 0.09) and 24 h (*p* = 0.03), as well as in cytobrush samples at 24 h (*p* = 0.04). Cytokine profiling revealed a trend toward decreased IL-1β at 6 h (*p* = 0.06) with a concurrent increase in IL-6 (*p* = 0.04) and a trend toward reduced IL-8 at 24 h (*p* = 0.09).

**Discussion:**

These findings demonstrate that resveratrol modulates specific components of the uterine inflammatory response in PBIE-susceptible mares. Such immunomodulatory effects may have relevance for improving reproductive efficiency, warranting further mechanistic and clinical investigation.

## Graphical abstract

**Figure:**
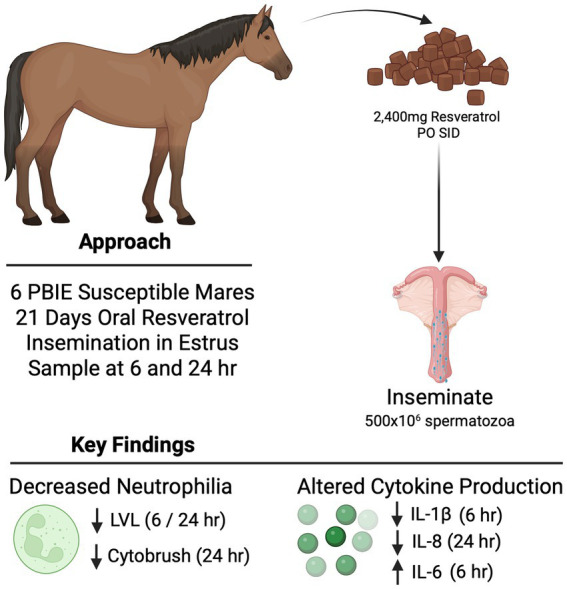

## 1 Introduction

The uterus experiences an innate immune response to the deposition of pathogens, semen, or even sterile solutions such as saline (1–3). If mares are unable to resolve this inflammation within 72 h, they are categorized as susceptible to the disease of persistent breeding-induced endometritis (PBIE), which is found in 10%–15% of the broodmare population (4). PBIE is associated with advanced age, poor endometrial quality, and a defective uterine immune system, which is primarily noted as a lack of anti-inflammatory signaling at 6 h after stimulus (5). This then leads to prolonged pro-inflammatory signaling and, therefore, immune cell chemotaxis to the uterine lumen (5–7). Mares susceptible to PBIE are predisposed to chronic uterine infections (8) and early embryonic loss (9) and are therefore deemed sub-fertile. This disease is considered the leading challenge in equine reproduction (10).

Resveratrol is a polyphenolic compound found in grapes, peanuts, and berries that has been associated with several health benefits (11, 12). This includes antioxidant, anti-carcinogenic, anti-viral, and anti-inflammatory properties (11). The impact of resveratrol on reproduction has been evaluated in other species and has been shown to improve ovarian function (13), reduce inflammation within the uterus (14), and overall improve fertility outcomes (15, 16). Additionally, resveratrol has been found effective in the treatment of various reproductive-related disorders, including polycystic ovarian syndrome (PCOS) (17), endometriosis (14), and preeclampsia (18, 19). Unfortunately, its impact on the uterus has not been assessed in horses.

In horses, resveratrol has been confirmed to exert anti-inflammatory and antioxidant effects on the immune system in tissues outside of the reproductive tract (20–23). *In vitro* treatment of peripheral lymphocytes with resveratrol was found to decrease the production of the pro-inflammatory interferon (IFN)-*γ* and tumor necrosis factor (TNF) (20). Additionally, resveratrol was found to alter equine neutrophil function by reducing the production of reactive oxygen species (ROS) in stimulated equine neutrophils, without affecting the degranulation of the stimulated neutrophils (22). In metabolic animals, resveratrol has been shown to decrease the production of both leptin and insulin (24). Resveratrol has also been found to reduce oxidative stress in horses, specifically by reducing the production of systemic malondialdehyde and glutathione peroxidase (23). Although two studies have evaluated the impact of resveratrol on sperm parameters (25, 26), no studies have investigated its impact on reproductive function in the mare.

An effective treatment for PBIE aims to improve the prolonged inflammation observed in susceptible mares by reducing pro-inflammatory mechanisms and their downstream effects within 24–36 h. Traditionally, PBIE has been treated with uterine lavage, ecbolics, anti-inflammatories, and antimicrobials (1). Alternative treatments that improve uterine clearance could reduce the need for antimicrobials, thereby improving the increasing prevalence of antimicrobial resistance (27). In this study, we propose to assess the efficacy of resveratrol as a treatment for PBIE, with the goal of improving breeding success by providing practitioners and breeders with a safe and effective option. Additionally, providing treatment strategies that may mitigate inappropriate use of antimicrobials could promote better antimicrobial stewardship in mares.

## 2 Materials and methods

### 2.1 General experimental procedure

#### 2.1.1 Classification of mares

A power analysis was conducted prior to the start of the trial using data from previous studies evaluating post-breeding uterine inflammation in mares susceptible to persistent breeding-induced endometritis (28–33). Based on these data, a sample size of six mares completing both a control and resveratrol-supplemented cycle provided ≥80% power to detect biologically relevant differences in neutrophilia and key inflammatory mediators at an alpha level of 0.05. The crossover design, in which each mare served as its own control, further increased statistical power by reducing the inter-animal variability inherent to reproductive immunology studies. Twelve mares with a documented clinical history of infertility were screened for susceptibility to PBIE, as previously described by Woodward et al. (5). This included a combination of poor endometrial quality, prolonged inflammation noted following a breeding challenge, and a history of subfertility. In brief, an endometrial biopsy was taken in diestrus and histologically assessed for (1) periglandular fibrosis, (2) inflammation, (3) glandular distribution, and (4) lymphatic lacunae. Susceptibility was determined based on endometrial biopsy scores, consistent with Kenney and Doig categories IIb to III (34). Additionally, mares were required to be clear of inflammation prior to insemination, as determined by negative culture and cytology. Prolonged inflammation after artificial insemination during estrus (>35 mm follicle, presence of uterine edema, and relaxed cervix) using 1×10^9^ freeze-killed sperm extended in a 30-mL EquiPro^®^ extender (MOFA Global, Verona, WI) for a minimum of 72 h post-breeding. This susceptibility was confirmed by intrauterine fluid accumulation at 72 h alongside positive cytology. A positive cytology was defined as greater than 2 PMNs observed for every 100 epithelial cells, and a positive microbial culture was defined as any bacterial growth after 24 h of aerobic culture. Six mares of mixed breeds and age (8–20 years) qualified for the study and were housed on dry lots with *ad libitum* access to water and minerals and supplemented with grass hay at the Colorado State University Equine Reproduction Laboratory in Fort Collins, CO. All experimental procedures were approved by the Institutional Animal Care and Use Committee of Colorado State University (Protocol number 5699).

#### 2.1.2 Resveratrol supplementation

All mares were housed under identical management and nutritional conditions throughout the treatment period, with *ad libitum* access to grass hay and water. Body weight and body condition score were recorded weekly to verify consistent feed intake and to monitor potential effects of treatment. Beginning on the day following ovulation, resveratrol was supplemented orally for one estrous cycle period (21.8 ± 0.08 days). Each mare received 2,800 mg resveratrol once daily, provided in a palatable pelleted vehicle to ensure complete consumption. Dosage was determined based on previous research, which found systemic alterations at this concentration (24). Complete intake was monitored, and any refusals were recorded. Control mares did not receive resveratrol. Adverse events were recorded, which included abdominal discomfort, diarrhea, anorexia, lethargy, or anaphylaxis.

#### 2.1.3 Insemination

Mares were examined daily via transrectal palpation and ultrasonography of their reproductive tracts for follicular development, endometrial edema, and uterine and cervical tones. When the presence of a pre-ovulatory follicle was noted (>35 mm), combined with reduced uterine tone, increased endometrial edema, and a relaxed cervix, mares were evaluated for the presence or absence of inflammation by endometrial cytology and aerobic cultures (35). Endometrial cytology was performed using a cytology brush, and uterine culture was performed with an endometrial swab (MOFA Global; Verona, WI, USA). Negative cytology was defined as the presence of less than two neutrophils per 100 epithelial cells, assessed at 400× magnification under light microscopy. Negative culture was defined as the complete absence of bacterial growth on a chromogenic agar plate at 24 h of incubation at 37 °C (Spectrum^®^ quad plate; Jorgensen Veterinary Supply; Loveland, CO; USA). Only mares clear of inflammation and infection were inseminated. If inflammation was observed, a rest cycle was implemented, and mares were treated accordingly. Over the course of two estrous cycles, mares were inseminated and received 1,500 IU of human chorionic gonadotropin (hCG; Intervet International B. V., Boxmeer, Netherlands) intravenously to standardize the interval between insemination and ovulation. Semen was previously collected from one stallion with a Missouri model artificial vagina (Nasco, Fort Atkinson, WI, USA) equipped with a gel filter (Animal Reproduction Systems, Chino, CA, USA). Sperm samples were adjusted to a concentration of 500 × 10^6^ spermatozoa in 30 mL lactated Ringer’s solution (LRS) and snap-frozen in liquid nitrogen three times before being stored at −80 °C.

#### 2.1.4 Evaluation of the innate immune response and sample collection

Innate immunity was measured as previously described (30). In brief, luminal fluid, neutrophilia, and inflammation were monitored at both 6 and 24 h after insemination. Fluid accumulation was measured as the largest circumference of retention. Subsequently, endometrial cytology was performed using a double-guarded cytology brush (MOFA Global, Verona, WI, USA). A low-volume lavage (LVL) was performed according to LeBlanc et al. (8). A uterine catheter was then passed through the cervix and into the uterus by an examiner wearing a sterile sleeve, and the cuff was inflated at the internal cervical os. The end of the catheter was attached to a bag containing 500 mL LRS, the entirety of which was infused into the body of the uterus. The uterus was then manipulated by rectal palpation for 30 s to distribute fluid throughout. The fluid was drained into a sterile bag by gravity flow. A resuspended aliquot of 40 mL was retrieved from the sample and centrifuged at 300×*g* for 10 min. All but 5 mL of the supernatant was decanted, and the pellet was resuspended in the remaining supernatant. Finally, 20 μL of the resuspended pellet was placed on a microscope slide to create a cellular smear. For endometrial cytology, cytobrushes were smeared on glass slides, which were then dried at room temperature, stained with Diff-Quik (Diff-Quik; Jorgensen Veterinary Supply; Loveland, CO; USA), and evaluated by light microscopy (400 magnification). Cytologic classification of the uterine biopsies and swabs was based on the number of polymorphonuclear neutrophils (PMN)s present per 100 endometrial epithelial cells examined (36).

#### 2.1.5 Cytokines

To measure the impact of resveratrol on uterine cytokine production, fluid from low-volume lavage was analyzed using an equine-specific multiple sandwich immunoassay based on flowmetric MILLIPLEX MAP^®^ technology, following the previously published workflow (Milliplex Equine Cytokine/Chemokine Panel; Millipore Sigma, Burlington, MA; USA) (37), to quantify IL-1β, IL-6, IL-8, IL-10, and TNF. Samples from LVL were measured undiluted, and standards prepared with LRS were added to all standards and quality controls. Antibody was washed prior to the addition of streptavidin. The means of intra- and inter-assay coefficients of variation were 2.7 and 3.7%, respectively. The detection level was defined as the signal-to-noise-ratio (limit of detection) divided by the square root of 2.

### 2.2 Statistics

Data were analyzed using SAS^®^ 9.4 (SAS Institute Inc., Cary, NC, USA). Prior to analysis, data were assessed for normality and equal variances using a Shapiro–Wilk and Bartlett’s test, respectively. Data fulfilled requirements for normality, and therefore, differences in fluid accumulation, neutrophilia, and cytokine concentrations were assessed using a general linear model with treatment as a random effect. Comparisons were made between group means, with *post hoc* comparisons performed using paired *t*-tests. A linear regression was utilized to assess the relationship between cytokine concentrations and neutrophilia. Significance was set at a *p*-value of <0.05 with statistical trends at *p* < 0.1. Data are presented as the mean ± the standard error of the mean.

## 3 Results

### 3.1 Clinical data

**Figure 1:**
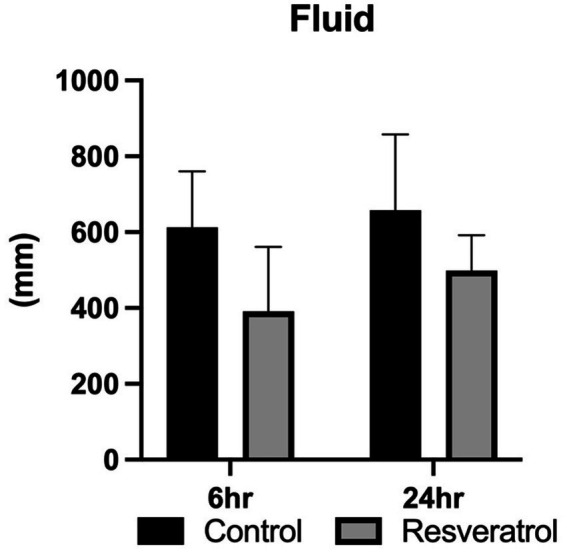
Fluid accumulation post-insemination in mares supplemented with resveratrol. No effect of time (*p* = 0.70) or resveratrol treatment (*p* = 0.69) was found to impact luminal fluid accumulation in mares susceptible to persistent breeding-induced endometritis. Data represent six mares (*n* = 6) evaluated across both control and resveratrol-supplemented cycles, with each mare serving as her own control.

No mares experienced adverse events following ingestion of resveratrol supplementation. Additionally, no refusals were recorded. All mares experienced significant uterine fluid accumulation following the deposition of freeze-killed semen into the uterus, regardless of supplementation (*p* = 0.35). No effect of treatment was noted at 6 (613.17 ± 147.1 mm vs. 391.0 ± 169.9 mm; *p* = 0.55) or 24 h following insemination (658.17 ± 199.5 mm vs. 498.8 ± 92.9 mm; *p* = 0.73; Figure 1) with regard to intraluminal uterine fluid retention. Only one mare required a rest cycle, and it was treated with amikacin to treat a *β*-streptococcus infection. The mare re-entered the trial once free of infection or inflammation, as determined by uterine culture and cytology.

**Figure 2:**
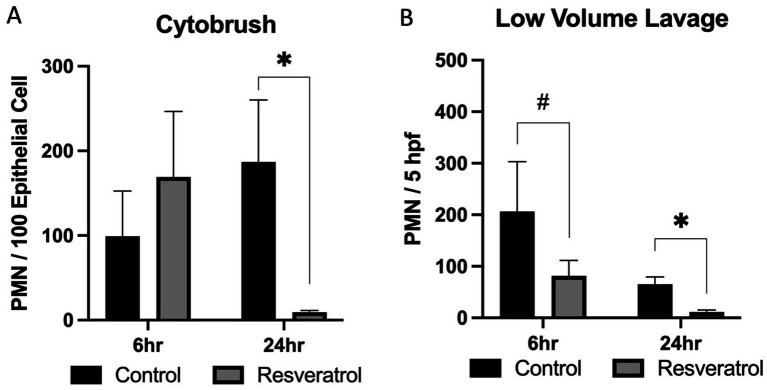
Neutrophilia of mares supplemented with resveratrol. When assessing cytobrush samples, there was no effect of either sampling time or resveratrol supplementation on neutrophilia. When assessing individual time points, no change in neutrophilia was noted at 6 h after insemination, but a significant reduction in neutrophilia was noted at 24 h **(A)**. When assessing low-volume lavage, a trend toward an effect of time was noted, but there was no overall effect of supplementation. When assessing individual time points, a trend toward a reduction in neutrophilia was noted at 6 h, and this reached significance at 24 h **(B)**. Neutrophil data were obtained from six PBIE-susceptible mares (*n* = 6) assessed at 6 and 24 h post-insemination during both control and resveratrol-supplemented cycles (within-mare crossover design). *p < 0.05, #*p* < 0.1.

When evaluating neutrophilia, both cytobrush and low-volume lavage were assessed. Neutrophilia was assessed via a double-guarded cytobrush and calculated as the ratio of PMN for every 100 endometrial epithelial cells observed at 20× under bright field microscopy. No effect of time on neutrophilia was noted (*p* = 0.55). As expected, immense neutrophilia was observed in the control group at 6 h following insemination (99 ± 53 PMN), and this was also observed in the resveratrol-supplemented mares at this time (168 ± 85 PMN). By 24 h following insemination, a significant reduction in neutrophilia was observed in the resveratrol-supplemented group when compared to the control cycle (187 ± 73 vs. 9 ± 2 PMN; *p* = 0.04; Figure 2A).

When assessing LVL fluid, neutrophilia was assessed as the number of PMNs observed within five high-power fields at 100× under bright field microscopy. A trend toward an effect of time (*p* = 0.06) was noted, as pronounced neutrophilia was noted at 6 h, and this decreased by 24 h following insemination. This was observed for both the control and resveratrol-supplemented groups. When comparing groups, a trend toward a decrease in neutrophilia was noted in the resveratrol-supplemented group at 6 h following insemination (206 ± 97 vs. 82 ± 30 PMN; *p* = 0.09). A decrease was also noted at 24 h when comparing control to supplemented groups (65 ± 14 vs. 12 ± 4 PMN; *p* = 0.03; Figure 2B).

### 3.2 Cytokines

**Figure 3:**
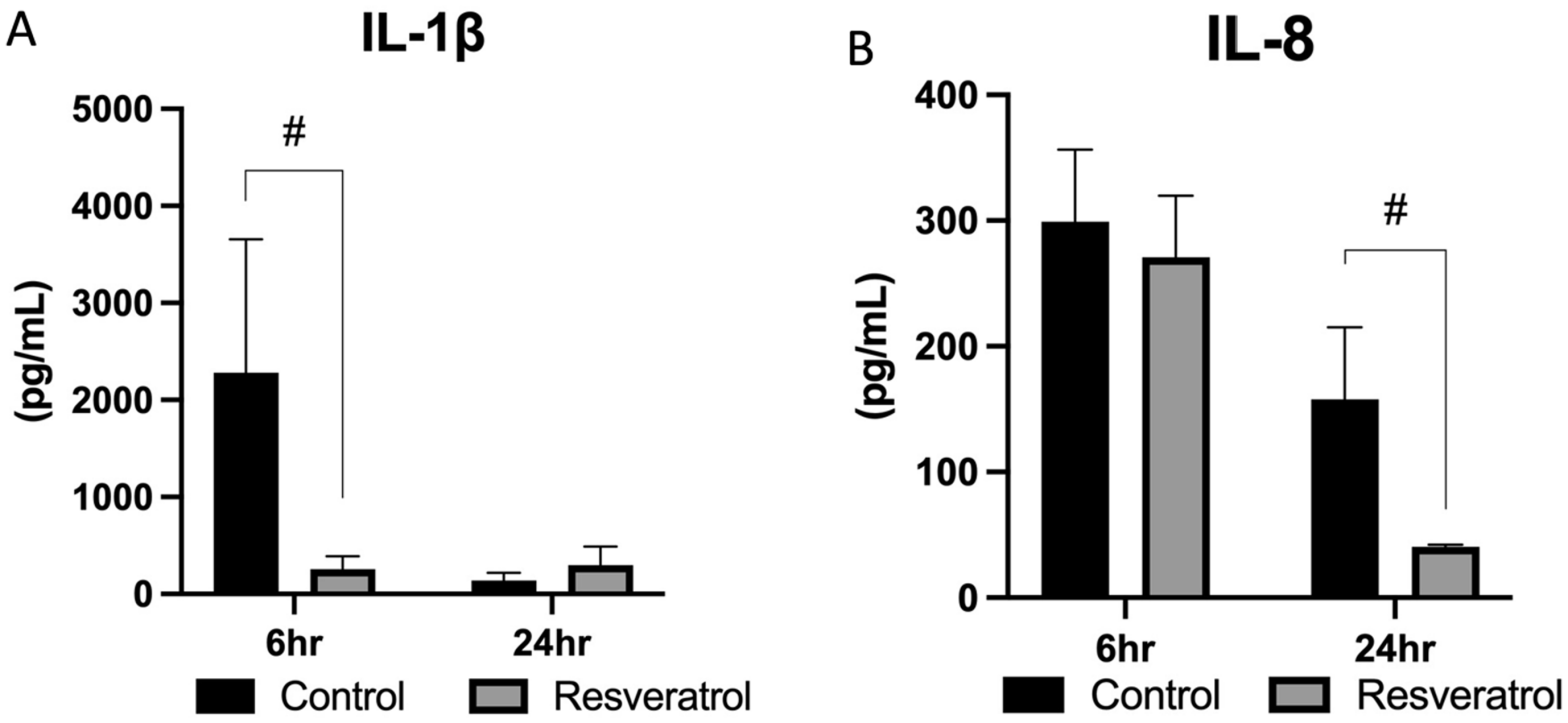
Production of pro-inflammatory cytokines following resveratrol supplementation. Cytokine concentrations were measured in low-volume lavage samples from six mares (*n* = 6) during control and resveratrol-supplemented cycles, with paired samples collected at each time point. Both IL-1β and IL-8 were reduced in the resveratrol-supplemented group. This was noted as a trend at 6 h following insemination for IL-1β **(A)** and at significant levels at 24 h for IL-8 **(B)**. *p < 0.05, #*p* < 0.1.

A number of cytokines were assessed within the luminal fluid collected during LVL, and this included pro-inflammatory IL-1β and IL-8, pleiotropic IL-6 and TNF, and anti-inflammatory IL-10. Neither TNF nor IL-10 were consistently above the limit of detection and were therefore not assessed. The initiator of inflammation, IL-1β, has been found to be imperative for detecting pathogenic material and foreign particles. Within the present study, no effect of time (*p* = 0.17) was noted. Heightened IL-1β was observed at 6 h in the control group, and this trended toward higher levels than that observed in the resveratrol-supplemented group (*p* = 0.06). By 24 h following insemination, concentrations of IL-1β were diminished for both control and supplemented groups, with no difference observed between the two (*p* = 0.53; Figure 3A).

Concentrations of IL-8 were assessed due to the chemotactic function of this pro-inflammatory cytokine. A significant effect of time was noted for IL-8 concentrations (*p* < 0.01), with uterine IL-8 decreasing over the course of time assessed. When evaluating the impact of resveratrol supplementation, no differences were noted at 6 h (*p* = 0.41), while a trend toward a decrease in IL-8 concentration was observed in the resveratrol-supplemented mares at 24 h after insemination (*p* = 0.09; Figure 3B).

**Figure 4:**
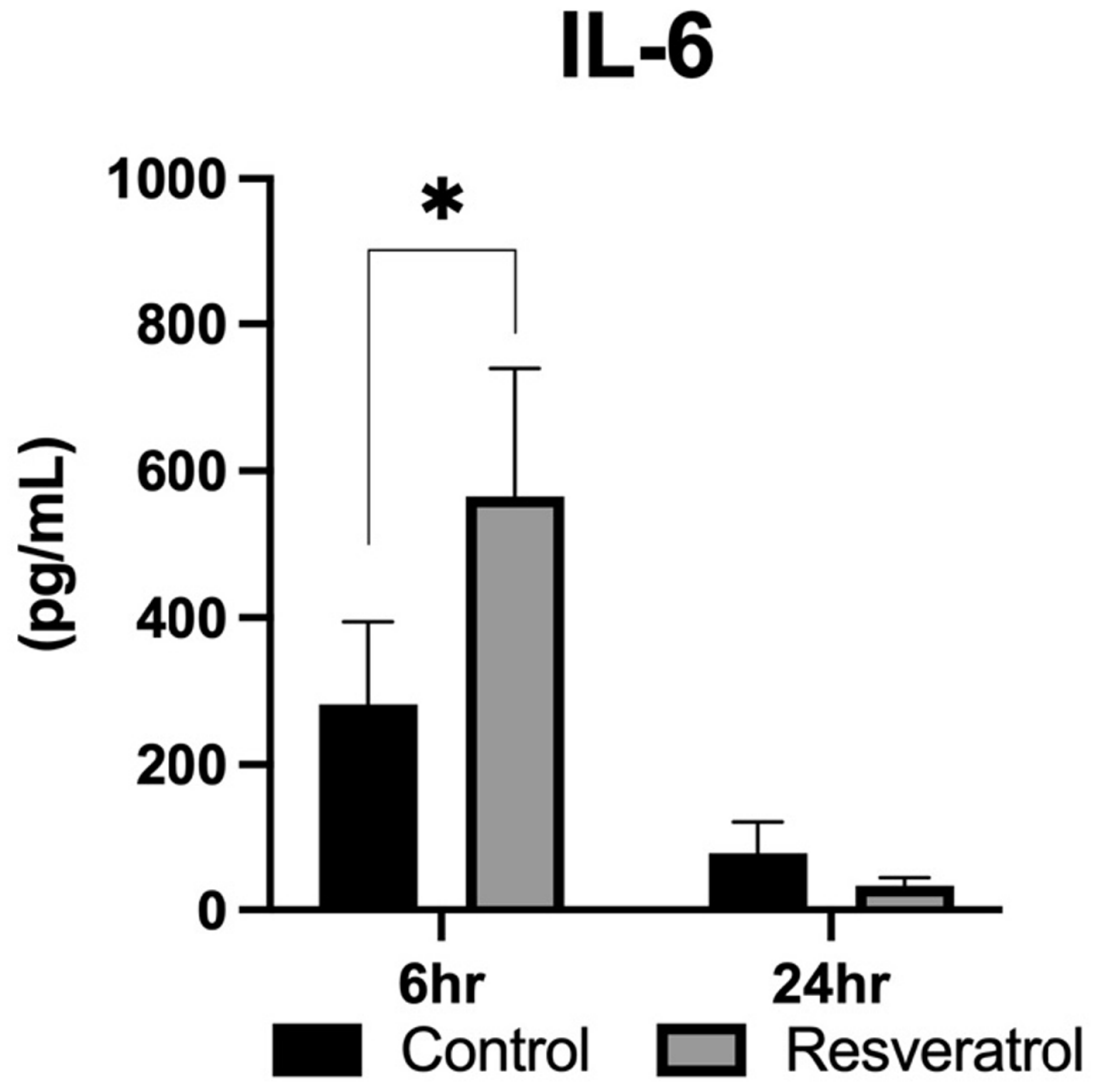
Production of pleiotropic cytokines following resveratrol supplementation. IL-6 concentrations reflect paired measurements from six mares (*n* = 6) evaluated at 6 and 24 h post-insemination in both treatment conditions. Intra-uterine IL-6 was significantly increased at 6 h while receiving resveratrol supplementation. No differences were noted at 24 h. *p < 0.05.

PBIE susceptible mares have been found to have a defective immune response to insemination, and this was specifically noted as a diminished production of pleiotropic (IL-6) and anti-inflammatory (IL-1RN, IL-10) cytokines at 6 h following insemination (5). In the present study, an effect of time was noted for the production of IL-6 (*p* < 0.01). This was noted as a dramatic decrease in luminal IL-6 by 24 h after insemination. When assessing individual time points, a significant increase in luminal IL-6 was noted in the resveratrol-supplemented group when compared to control cycles (*p* = 0.04). By 24 h following insemination, no differences were noted when comparing the two groups (*p* = 0.32; Figure 4).

## 4 Discussion

Persistent breeding-induced endometritis (PBIE) is the leading cause of infertility in equine reproduction (10). Treatment for this disorder is usually multi-modal, costly, and requires veterinary intervention to be successful. In this study, we have demonstrated a non-invasive daily oral supplement that improved markers of inflammation following insemination in mares susceptible to PBIE. This included a reduction in uterine neutrophilia, a reduction in pro-inflammatory cytokine production, and an increase in key inflammatory mediators that are essential for the resolution of this persistent inflammation. To our knowledge, we are the first to identify an oral supplement that may benefit this population of horses.

The disorder of PBIE affects approximately 15% of all broodmares (4), with considerable financial impact to breeders. The pathophysiology of this disease has been elegantly investigated by multiple groups, with key focuses on mechanical clearance, immune deficits, and prolonged inflammatory mechanisms (2, 3, 6, 8, 9, 38). Woodward et al. (5) found PBIE susceptible mares to fail in their transition from a pro-inflammatory to an anti-inflammatory phenotype within 6 h after insemination. This dysregulation is typified by impaired induction of anti-inflammatory and pleiotropic cytokines such as IL-6, IL-1RN, and IL-10, which leads to persistent chemotaxis of neutrophils into the uterine lumen (3, 5, 6, 39). This process is believed to be the central driver of PBIE-associated infertility in mares. In the present study, resveratrol supplementation attenuated early neutrophil influx and shifted cytokine production in a direction suggestive of improved control or suppression of inflammation. The observed reduction in luminal IL-1β at 6 h is particularly notable, as IL-1β is a central amplifier of uterine inflammation, promoting both chemokine production and neutrophil migration (40). This decrease in IL-1β at 6 h was mimicked by a decrease in neutrophilia noted within LVL, further indicating the clinical impact of this suppression. A decrease in IL-8 was also noted, albeit at 24 h after insemination. IL-8 is the primary chemotactic signal for equine neutrophils (41), further supporting the conclusion that resveratrol mitigates the excessive inflammatory recruitment characteristic of susceptible mares.

Importantly, resveratrol increased luminal concentrations of IL-6 at 6 h post-insemination. While IL-6 is often labeled pro-inflammatory, numerous studies have identified downstream anti-inflammatory impacts of this cytokine; thereby identifying the pleiotropic function of IL-6 (42). This has also been confirmed in horses (43). In mares resistant to PBIE, IL-6 rises within 2 h following insemination and plays an anti-inflammatory role in transitioning the endometrium toward repair and immunomodulation (5, 6). Conversely, PBIE-susceptible mares fail to produce adequate IL-6 during this critical time (5). Thus, the IL-6 elevation observed in resveratrol-supplemented mares at 6 h may indicate restoration of physiologic immune signaling rather than exacerbation of inflammation. Mechanistically, resveratrol is known to influence IL-6 expression and production, and this is impacted by time of sampling, concentration of resveratrol supplemented, species investigated, and disease (44–47). Taken together, these cytokine data may indicate that resveratrol modifies the susceptible mare’s inflammatory response to mimic what has been described in the resistant animal. This includes dampening excessive pro-inflammatory activation while supporting early regulatory cytokine production.

Tumor necrosis factor (TNF) and interleukin-10 (IL-10) concentrations in uterine luminal fluid were consistent below the limit of detection for the multiplex immunoassay and, therefore, were not included in statistical analyses. To our knowledge, limited data are available regarding the presence of cytokines in uterine fluid obtained by low-volume lavage, and this is further confounded by disease, timing of sampling, and low-volume lavage technique (48, 49). Therefore, it is hard to compare this study with previous reports of TNF and IL-10 within the equine uterine lumen low-volume lavage fluid following breeding. Although the assay employed is highly sensitive and validated for equine cytokines (37, 43, 50–52), the dilutional nature of low-volume lavage and the rapid kinetics of these cytokines during endometritis likely limited their detectability at the time points evaluated. As such, the absence of quantifiable TNF and IL-10 should not be interpreted as a lack of biological relevance, but rather reflects technical and temporal constraints inherent to luminal cytokine assessment in the mare.

The reduction in neutrophilia observed at both 6 and 24 h further supports this interpretation. Excessive or persistent neutrophilic infiltration contributes to tissue damage, delayed resolution, and impaired uterine clearance in PBIE (1, 53, 54). *In vitro* studies in horses have shown that resveratrol decreases ROS, alters myeloperoxidase activity, and attenuates pro-inflammatory cytokine production (22), all of which are consistent with a broad anti-inflammatory and immunomodulatory phenotype. Additionally, resveratrol has been found to decrease neutrophil influx in other disease models, including airway inflammation (55), lupus (56), and rheumatoid arthritis (57). Similar suppression of neutrophilia has also been noted within the reproductive tract, including during endometriosis (58), PCOS (59), preeclampsia (60), and pre-term birth (61), which has led to increased usage of this polyphenol to improve reproductive outcomes in the human. The *in vivo* findings within the present study extend this understanding to the reproductive tract of the horse and are among the first to demonstrate that resveratrol’s immune modulation can be harnessed clinically within the mare.

We are unsure why the cytology data differed between the two sampling techniques, but this is a common finding in equine reproduction (62–64). A cytobrush and an LVL sample have fundamentally different microenvironments within the uterus, which can lead to varying and inconsistent diagnostic results. A *cytobrush* collects cells directly from the *superficial endometrium*, capturing adherent neutrophils, epithelial cells, and early inflammatory changes that may not yet be shed into the lumen. In contrast, LVL samples the *luminal fluid*, which tends to dilute cellular material and preferentially reflects inflammation that has progressed enough to shed cells or accumulate secretions. There are varying opinions on which technique provides the greatest sensitivity and specificity to diagnose uterine inflammation (62, 65), but both are capable of assessing neutrophilia and are commonly used in clinical practice.

From a clinical standpoint, these mechanistic improvements align with the therapeutic goals for PBIE management, as resveratrol supplementation was found to decrease inflammatory cell influx, improve timely immune resolution, and enhance physiologic uterine clearance. While uterine fluid accumulation was not altered by resveratrol in this study, PBIE must be considered multifactorial, and immune dysregulation may precede or operate independently of fluid retention. Additionally, luminal fluid measurements are notoriously inaccurate and difficult to quantify. The reduction in neutrophilia and shifts in cytokine signaling observed in this study provide evidence that resveratrol may improve the underlying immune environment that predisposes mares to be susceptible to this persistent inflammation. Importantly, resveratrol represents a non-antibiotic, non-hormonal strategy that could reduce reliance on repeated ecbolics, lavage, and antimicrobials—interventions that carry labor costs, practitioner time, and implications for antimicrobial stewardship.

Nevertheless, several limitations must be considered. The relatively small number of animals limits the detection of subtle effects on clinical outcomes. Additionally, the study evaluated a single dose and formulation of resveratrol, and the authors acknowledge that a dose response is necessary. A previous study on resveratrol in other species has investigated a broad range of doses (1 mg/kg–300 mg/kg) (11, 15, 16, 66), and therefore extrapolations made between studies and species are difficult. Additionally, although multiple studies have been performed on the impact of resveratrol in the horse (20, 22, 24, 26), we selected a dose that has been previously described as altering systemic endpoints (24), but not within the uterus. Therefore, the impact of this dose on the pharmacokinetics of the equine reproductive tract remains incompletely described. In addition to mechanistic immune endpoints, future studies should directly evaluate fertility outcomes, including pregnancy establishment, early embryonic loss, and foaling rates, to determine the clinical relevance of resveratrol supplementation in PBIE-susceptible mares. Such a study will be essential to define whether modulation of the post-breeding uterine immune environment translates into sustained improvements in reproductive efficiency under field conditions, in addition to the safety of oral resveratrol supplementation during equine pregnancy.

In conclusion, resveratrol supplementation modulates early uterine immune responses in mares susceptible to PBIE, characterized by reduced neutrophilia, diminished IL-1β and IL-8 production, and an increase in early IL-6 signaling. These changes align mechanistically with the clinically relevant reduction of neutrophilia alongside a restoration of the immune pathways known to be defective in susceptible mares (5). While additional research on fertility and pregnancy maintenance is warranted, these findings highlight resveratrol as a promising immunomodulatory therapy that addresses core biological drivers of PBIE and offers a clinically relevant, non-invasive, and mechanism-based approach to improve post-breeding uterine health.

## Data Availability

The data that support the findings of this study are openly available in Dryad at DOI: 10.5061/dryad.tx95x6bbs.
